# Interferonopathies masquerading as non-Mendelian autoimmune diseases: pattern recognition for early diagnosis

**DOI:** 10.3389/fped.2023.1169638

**Published:** 2023-08-09

**Authors:** Samuel Gagne, Vidya Sivaraman, Shoghik Akoghlanian

**Affiliations:** ^1^ Division of Pediatric Rheumatology, Nationwide Children’s Hospital, Columbus, OH, United States; ^2^ Department of Pediatrics, The Ohio State University, Columbus, OH, United States

**Keywords:** early-onset, vasculitis, vasculopathy, interferonopathy, autoinflammatory, monogenic lupus

## Abstract

Type I interferonopathies are a broad category of conditions associated with increased type I interferon gene expression and include monogenic autoinflammatory diseases and non-Mendelian autoimmune diseases such as dermatomyositis and systemic lupus erythematosus. While a wide range of clinical presentations among type I interferonopathies exists, these conditions often share several clinical manifestations and implications for treatment. Presenting symptoms may mimic non-Mendelian autoimmune diseases, including vasculitis and systemic lupus erythematosus, leading to delayed or missed diagnosis. This review aims to raise awareness about the varied presentations of monogenic interferonopathies to provide early recognition and appropriate treatment to prevent irreversible damage and improve quality of life and outcomes in this unique patient population.

## 1. Introduction

First discovered in the 1950 s (1), interferons (IFNs) are a large group of signaling molecules that promote and regulate innate immune system responses to viral infection. IFNs are divided into three major classes (Type I, II, and III), each with distinct roles within the immune system. Type I IFNs (IFN-1) are predominantly made up of IFN-α and IFN-*β* and are expressed by many cell types. IFN-1 gene expression can be induced by cytokines, IFNs themselves, and pattern recognition receptors such as toll-like receptors and cytosolic nucleic acid receptors such as MDA5 and RIG-I (2). IFN signaling proceeds via the Janus kinases (JAK)-signal transducer and activator of transcription proteins (STAT) pathway upon receptor binding, ultimately transcribing several pro-inflammatory IFN-stimulated genes (3).

Type I interferonopathies are a broad category of conditions associated with increased IFN-1 gene expression (4). Initially, interferonopathy was limited to a group of Mendelian disorders caused by a genetic defect that disrupts homeostatic control and leads to IFN-mediated responses. However, the term has been extended to non-Mendelian autoimmune diseases such as dermatomyositis, systemic sclerosis, and systemic lupus erythematosus (SLE), as well as more rare diseases such as Degos disease, where IFN signaling is important to pathogenesis (5–7). Type I interferonopathies share several clinical manifestations and treatment implications. Specifically, they frequently affect the central nervous system (CNS) and skin but are not limited to these systems (8, 9). An elevated peripheral blood IFN signature is not required but can be helpful for diagnosis (10). Unfortunately, few centers check IFN signatures, and there are no internally standardized methodologies or references.

Glucocorticoids, conventional disease-modifying anti-rheumatic drugs (DMARDs) such as methotrexate, mycophenolate mofetil (MMF), and even bone marrow transplantation (BMT) have historically been used to treat interferonopathies. More recently, biologic DMARDs to target cytokines and/or small-molecule DMARDs to target JAKs have become an increasingly common therapeutic strategy. The US Food and Drug Administration's Compassionate Use Program was initiated in 2011 to treat pediatric patients with rare Mendelian autoinflammatory diseases that have IFN-mediated pathology (3, 11). Subsequently, several case series have been published demonstrating the potential efficacy of JAK inhibitors in treating such diseases (12, 13). However, an increased risk of serious and opportunistic infections has been observed and BK viremia, nephropathy, and, rarely, progressive multifocal leukoencephalopathy have been reported with JAK inhibitor use (14, 15).

Patients with monogenic interferonopathies often present within the first week of life; however, late onset in the teenage years has been reported. Patients may share similar symptoms with more common non-Mendelian autoimmune diseases (see Table 1), leading to a delayed or missed diagnosis (Table 1). This review aims to bridge the gap between autoimmunity and monogenic interferonopathies to enable early recognition and diagnosis and improve outcomes in this unique patient population.

**Table 1:** Genetics and disease characteristics of monogenic type I interferonopathies.

Disease Name	Genetics	Disease characteristics	Diseases with similar presentation
SAVI	Autosomal dominant gain of function mutation in *TMEM173*, encoding STING	Arthritis, ILD, neurological manifestations, dermal small vessel vasculitis, Raynaud's/chilblains	ANCA-associated vasculitis
COPA Syndrome	Autosomal dominant mutation in *COPA*	Glomerulonephritis, arthritis, ILD, immune dysfunction, pulmonary capillaritis	ANCA-associated vasculitis, SLE
PRAAS/CANDLE	Autosomal recessive loss of function mutations in the genes encoding proteasome components which constitute or regulate the ubiquitin/ proteasome system, including *PSMB8, PSMB4, PSMB8*, and *PSMB9*	Fever, neutrophilic dermatosis, lipodystrophy, myositis, pulmonary hypertension, small vessel vasculitis	ANCA-associated vasculitis
Monogenic SLE	Varies, notable mutations include early complement deficiencies (C1q, C1r/C1s, C2 and C4) as well as mutations in *TREX1*, *SAMHD1*, *ADAR1*, *IFIH1*, *RNASEH2A*, *RNASEH2B*, and *RNASEH2C*	Glomerulonephritis, cytopenias, leukocytoclastic vasculitis, neurologic involvement including encephalopathy, cerebral vasculitis, seizures, focal motor deficits	SLE, dermatomyositis
DADA2	Autosomal recessive loss of function mutation in adenosine deaminase 2	Fever, immune dysfunction, cytopenia/bone marrow failure, medium-vessel vasculitis, early onset-stroke	Polyarteritis nodosa
STAT1 Gain-of-Function	Autosomal dominant gain of function mutation in *STAT1*	Mucocutaneous candidiasis, recurrent sinopulmonary infections, autoimmune endocrinopathies, cytopenias, inflammatory bowel disease, CNS aneurysms and large vessel vasculitis, predominantly involving aorta	Behcet's, Takayasu's arteritis, SLE

AGS, Aicardi-Goutières syndrome; ANCA, antineutrophil cytoplasmic antibody; CNS, central nervous system; COPA, Coatomer subunit alpha; DADA2, deficiency of adenosine deaminase 2; ILD, interstitial lung disease; PRAAS/CANDLE, Proteasome-associated autoinflammatory syndromes/chronic atypical neutrophilic dermatosis with lipodystrophy and elevated temperature; PSMB, proteasome subunit beta; SAVI, STING-associated vasculopathy; SLE, systemic lupus erythematosus; STAT1, signal transducer and activator of transcription 1.

## 2. STING-associated vasculopathy with onset in infancy (SAVI)

Stimulator of IFN genes (STING)-associated vasculopathy with onset in infancy (SAVI) is a rare autoinflammatory disease caused by gain-of-function (GOF) mutations in *TMEM173* which encodes the STING transmembrane protein (16). STING is involved in the innate immune response, functioning as an adapter molecule of the cytosolic DNA danger signal-sensing machinery. In SAVI, mutations in STING lead to its sequestration from the endoplasmic reticulum Golgi intermediate compartment, leading to excessive expression of interferon stimulated genes (17). Children with SAVI typically present in early childhood with dermal small vessel vasculopathy and microangiopathic thrombosis that varies in severity from chilblain lesions and Raynaud's phenomenon to severe progressive tissue loss involving the earlobes, nose, and digits. Progressive interstitial lung disease (ILD) with or without pulmonary hypertension may be present in severe disease. Pulmonary manifestations are typically detected via chest CT scan and spirometry, although lung biopsy may be required to differentiate infectious from inflammatory disease (18, 19). Myositis and autoantibody production are common in SAVI, thus misdiagnosis of SLE or juvenile myositis is not uncommon. In SAVI, autoantibodies are not associated with disease severity but instead are likely modulated by genetic factors (19). Neurological manifestations are rare compared to other interferonopathies, and developmental delays have not been reported in children with SAVI (20, 21). Arthritis was observed in one-third of SAVI patients and can be destructive (16). SAVI can be challenging to diagnose as many patients have normal laboratory parameters, including complete blood counts and inflammatory markers. Therefore, it is crucial to consider the diagnosis of SAVI in young patients with arthritis and SLE-like symptoms. Treatment with different JAK inhibitors (ruxolitinib, baricitinib, and tofacitinib) has resulted in improvement in the lung disease, significant decrease in vasculitis flares, and prevention of progression of spontaneous amputation in patients with SAVI (13).

## 3. Coatomer subunit alpha (COPA) syndrome

Coatomer subunit alpha (COPA) syndrome is a rare autosomal dominant interferonopathy caused by *COPA* gene mutations. COPA is part of COPI and COPII (coat member protein complex I and II) that are involved in bidirectional vesicular trafficking between the endoplasmic reticulum and the Golgi complex. *COPα* mutations cause dysfunctional protein binding and retrograde transport through the COPI complex (22). It has been hypothesized that this aberrant protein trafficking leads to endoplasmic reticulum stress via the unfolded protein response, leading to subsequent autoimmunity (23). However, recent research suggests that *COPA* mutations result in STING overexpression with chronic IFN-1 activation and autoimmunity. Specifically, defects in *COPA* result in an inability to traffic and degrade STING. The result is multimerization of STING which becomes spontaneously active even without the STING ligand, leading to chronic IFN-1 activation (24–26).

The presentation of COPA syndrome is clinically similar to SAVI and antineutrophil cytoplasmic antibody (ANCA)-associated vasculitis although early age of presentation assists in differentiating COPA from ANCA-associated vasculitis. COPA syndrome presents in young children with features that can include pulmonary symptoms, renal disease, arthritis, and immune dysregulation (27). Pulmonary manifestations are variable but most commonly present as progressive pulmonary hemorrhage secondary to pulmonary capillaritis (28). Chest CT scans often demonstrate diffuse ground glass opacities at the onset with eventual pulmonary nodule development and cystic changes (27, 29). Untreated, nearly all patients with COPA syndrome develop ILD, with lymphocytic interstitial pneumonia and follicular bronchitis being the most common (28, 30, 31). Renal involvement in COPA syndrome occurs in approximately 44% of patients, typically in the second decade of life (27). Renal involvement in most patients presents with glomerulonephritis with proteinuria and hematuria which can mimic ANCA-associated vasculitis or SLE (32). The histologic manifestations vary but include crescentic disease (with or without immune deposits), focal segmental glomerulosclerosis, membranous nephropathy, and IgA nephropathy. “Most (95%) patients have musculoskeletal involvement. Musculoskeletal involvement typically presents in the second decade of life as non-erosive polyarthritis although it can be the presenting symptom, leading to a misdiagnosis of juvenile idiopathic arthritis. Osteonecrosis has also been reported (27, 30). Immune dysregulation is frequently observed in 80% of patients with antinuclear antibodies, rheumatoid factor, anti-cyclic citrullinated antibodies, c-ANCA, and p-ANCA antibodies. CD4+ T-cells also frequently skew towards a Th17 phenotype. Cytopenia or hypogammaglobulinemia are not typically present (27). Less frequently, involvement of other systems, such as CNS, liver, thyroid, and skin, have been reported (30, 33). No standard treatments for COPA syndrome exist. Typically, systemic corticosteroids are given in combination with other immunosuppressants such as MMF, rituximab, and hydroxychloroquine (27, 28, 34). Successful treatment with JAK inhibitors has also been reported (35, 36).

## 4. STAT1 gain-of-function

STAT1 Gain-of-Function (GOF) is an autosomal dominant disorder caused by several mutations that lead to STAT1 hyperphosphorylation, subsequent autoimmunity, and increased susceptibility to infection, notably chronic mucocutaneous candidiasis (37). Usually, STAT1 exists as a cytosolic transcription factor downstream of IFN and IL-27 receptors. Upon receptor binding, STAT1 is phosphorylated, forms either a heterotrimer or homodimer, and migrates to the nucleus to regulate gene expression (38). In STAT1 GOF, impaired dephosphorylation or high STAT1 levels activate pathways, decrease Th17 serum levels, and increase expression of interferon stimulated genes (37–39). These changes increase susceptibility to infections and autoimmunity; however, the exact mechanisms are unknown.

The presentation of STAT1 GOF disease is very heterogenous. However, increased susceptibility to infection is universally seen. Over 98% of patients with STAT1 GOF develop chronic mucocutaneous candidiasis which can help differentiate the diagnosis from other autoimmune diseases. Increased sinopulmonary and mucocutaneous bacterial and viral infections are also commonly observed. Autoimmune manifestations vary and occur in approximately one-third of patients. Autoimmune endocrinopathies such as thyroid disease and type I diabetes mellitus are most common. However, SLE, autoimmune skin conditions, inflammatory bowel disease, and autoimmune cytopenias can also occur (40). Vascular involvement has been well described in STAT1 GOF and predominantly presenting as cerebral aneurysms (38, 41), although large-vessel vasculitis that can mimic Takeyasu's arteritis has also been reported (42–44).

Additionally, with predominant recurrent aphthous ulcers at presentation, several patients were misdiagnosed with Behcet's disease (45, 46). Given the increased infection susceptibility and risk of autoimmunity, STAT1 GOF treatment is challenging and can include antimicrobial and immunosuppressive therapy. JAK inhibitors have emerged as a promising treatment for patients with STAT1 GOF, although many still ultimately require BMT (47, 48).

## 5. Proteasome-associated autoinflammatory syndrome/chronic atypical/neutrophilic dermatosis with lipodystrophy and elevated temperature (PRAAS/candle)

Proteasome-associated autoinflammatory syndrome (PRAAS)/chronic atypical neutrophilic dermatosis with lipodystrophy and elevated temperature (CANDLE) is a rare autosomal recessive interferonopathy caused by loss-of-function mutations in the genes encoding proteasome components that either constitute or regulate the ubiquitin/proteasome system (*PSMB8, PSMA3, PSMB4, PSMB8,* and *PSMB9*) and lead to uncontrolled IFN-1 activation. Clinically, early-onset neutrophilic dermatosis with mononuclear interstitial infiltrate appears to be pathognomonic for PRAAS/CANDLE (49). Comparable to other interferonopathies, vasculopathy, perivascular infiltrates, and neutrophilic vasculitis can be observed, and a case of ANCA-associated vasculitis has been reported (50). Self-limited attacks of myositis are frequently observed in patients with PRAAS/CANDLE. However, unlike juvenile dermatomyositis, the muscle MRIs in PRAAS/CANDLE shows patchy distribution of muscle inflammation (12). Pulmonary manifestations, such as pulmonary hypertension, can be life-threatening. Unlike SAVI and COPA, lung disease rarely progresses to ILD in PRAAS/CANDLE. Pulmonary function tests and echocardiograms are recommended for screening and monitoring. Patients with PRAAS/CANDLE may also present with neurologic features such as headaches and aseptic meningitis. Lumbar puncture typically reveals sterile cerebrospinal fluid with pleocytosis. During PRAAS/CANDLE disease flares, laboratory studies show cytopenia, nonspecific hypochromic or normocytic anemia, increased acute-phase reactant levels, elevated transaminases, and abnormal muscle enzymes (49). A child with atypical myositis and cutaneous rash who fails to respond to standard treatment regimens should raise concerns about the possibility of an underlying interferonopathy such as PRAAS/CANDLE. PRAAS/CANDLE patients are also at higher risk of developing metabolic syndrome, especially with the use of glucocorticoid therapies. The JAK inhibitor baricitinib has been used successfully with reported reduction in daily symptoms and glucocorticoid requirements (12).

## 6. Monogenic systemic lupus erythematosus (SLE)

SLE is a chronic multisystem inflammatory disease characterized by elevated IFN-1 activity in approximately half of patients. Monogenic or early-onset SLE is strongly linked to genetics and known SLE-associated risk alleles, whereas the more common (“classic”) SLE that presents in older children and adults is thought to be more multifactorial in etiology. Sex differences are less pronounced in monogenic SLE in comparison to classic SLE. Over 100 susceptibility loci for SLE and 35 genes for monogenic SLE and SLE-like disease have been discovered by genome-wide association studies (51, 52). Younger age at presentation of SLE predicts damaging variants in genes that cause monogenic SLE regardless of consanguinity, ancestry, sex, or the prevalence of individual clinical features (53).

Monogenic SLE was first described in the 1970s and found to be caused by early complement deficiencies (C1q, C1r/C1s, C2, and C4). While complement pathway defects continue to be the most common subcategory of monogenic SLE, the second leading cause of monogenic SLE is in mutation in genes related to IFN regulation. Autosomal recessive *TREX1* gene defects have recently been linked to SLE, familial chilblain lupus, and Aicardi-Goutières syndrome (AGS) (54). *TREX1* deficiency leads to the accumulation of cytosolic DNA fragments that activates the innate DNA sensing response, resulting in IFN overproduction. Mutations in genes related to IFN regulation such as *SAMHD1*, *ADAR1*, *IFIH1*, *RNASEH2A*, *RNASEH2B*, and *RNASEH2C* have also been identified as causing familial chilblain lupus and AGS (55–58).

AGS presents early in infancy, often with chilblains. Neurologic manifestations are prominent in patients with AGS, such as early-onset encephalopathy, basal ganglia or white matter calcifications, focal motor findings, and seizures. Unlike the previously discussed interferonopathies, progressive neurologic impairment is common in AGS. Neuroimaging is required at baseline and follow-up to evaluate CNS vasculopathy, which is often referred to as Moyamoya disease. Cerebral vasculitis can mimic early AGS and lead to delays in diagnosis and likely worse outcomes. Similar to non-Mendelian SLE, autoantibodies can be present in monogenic SLE and AGS, along with cytopenias (e.g., anemia and thrombocytopenia) and elevated transaminases. Elevation in transaminases may be due to hepatic involvement or myositis. Cardiac involvement is rarely observed in AGS.

About 90%–95% of children with monogenic SLE generally satisfy the 2019 European League Against Rheumatism/American College of Rheumatology (EULAR/ACR) and 2012 Systemic Lupus International Collaborating Clinics Classification criteria. Positive antinuclear antibodies with malar rash are the most common clinical features, occurring in 97% of children with monogenic SLE (53, 59). Glomerulonephritis is reported with unclear frequency and is often partially responsive to conventional immunosuppressive medications (60). IFN-1 contributes to glomerular injury through direct damage to kidney cells, induction of autoantibody generation and immune complex deposition, and inflammatory cell chemotaxis (61). The most frequent renal biopsy pattern is proliferative nephritis. However, vascular changes, such as thrombotic microangiopathy, have been reported (62). Glucocorticoids are effective. Often patients with monogenic SLE have limited response to classic SLE therapy like hydroxychloroquine, azathioprine, MMF, and cyclophosphamide. With the fundamental discoveries about pathogenesis of interferonopathies, multiple clinical trials are currently being conducted to evaluate the efficacy and safety of JAK inhibitors.

## 7. Deficiency of adenosine deaminase-2 (DADA2)

DADA2 is an autosomal recessive autoinflammatory disorder caused by mutations in the *ADA2* gene (63, 64). The ADA2 enzyme is primarily found extracellularly and regulates the innate immune response. The exact mechanism by which this mutation leads to systemic inflammation is unknown. However, it has been suggested that a disruption in extracellular adenosine regulation leads to the generation of neutrophil extracellular traps (NETs). NET formation leads to macrophage activation and subsequent release of TNF-α (65, 66). Recently, patients with active DADA2 also demonstrated an IFN-1 signature that may be correlated with disease activity (67, 68). Multiple mechanisms have been proposed to explain the increased IFN-1 expression, but the significance of this increase, and its interaction with TNF-α, remains unclear (69, 70).

**Figure 1:**
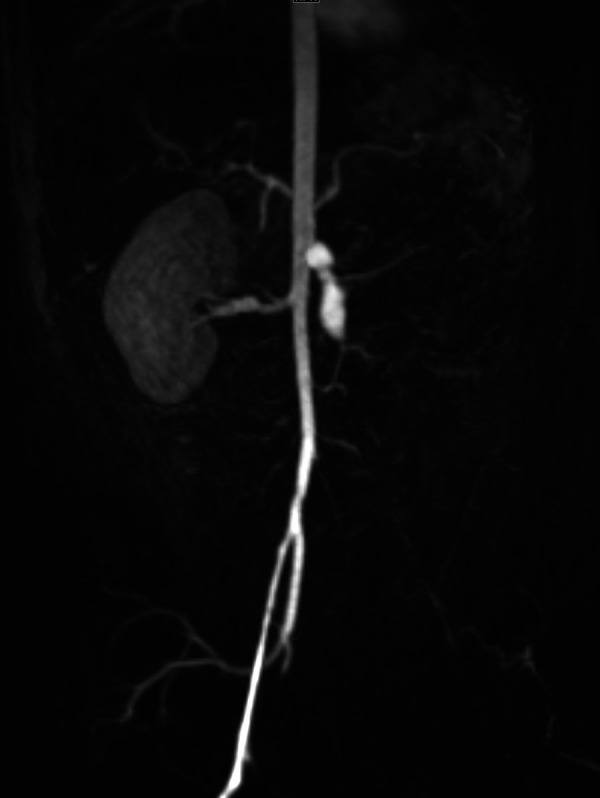
Renal infarct in an infant with DADA2. Severe narrowing and proximal left common iliac artery occlusion at the bifurcation. Fusiform, aneurysmal dilation of the mid to distal superior mesenteric artery segment. Renal artery and kidney are absent.

Clinically, DADA2 has a very similar presentation to polyarteritis nodosa (PAN) with fever, medium-vessel vasculitis, livedo racemosa, and early-onset stroke (71). Although neurologic and skin involvement is the most common, any organ system can be affected. Gastrointestinal, renal (Figure 1), cardiopulmonary, and musculoskeletal involvement can occur as well (72). Patients with DADA2 may display a degree of immune dysregulation, although this symptom is variable and strongly associated with genotype. Immune dysregulation may be limited to mild hypogammaglobulinemia and cytopenias but may be as severe as pure red cell aplasia and bone marrow failure (73, 74). While early age of onset can assist in differentiating DADA2 from PAN, some cases of infantile PAN may not have DADA2 mutations and some cases of DADA2 may not present until the second decade of life (74). Thus, it is reasonable to consider DADA2 testing in pediatric patients with symptoms consistent with PAN, especially if they have symptoms of immune dysregulation that would be more atypical in PAN. TNF inhibitors are a very effective treatment for vasculitis-predominant phenotypes (75, 76). JAK inhibitors may also be effective (77). Notably, severe hematological manifestations tend to be resistant to typical treatments and often require BMT (73).

## 8. Discussion

Type I interferonopathies are a group of heterogeneous diseases associated with increased IFN-1 stimulated gene expression. Many of the monogenic interferonopathies mimic more common autoimmune diseases such as SLE, juvenile myositis, PAN-, ANCA-, and CNS-vasculitis. Given the significant overlap in clinical features among these disorders, a high index of suspicion and genetic evaluation to confirm the specific disease are crucial for diagnosis and management. As testing methods such as next-generation sequencing—including targeted gene panels and whole exome or whole genome sequencing—have become more affordable and accessible over recent years, screening for pathogenic variants is now recommended by EULAR/ACR (78).

Including such diverse diseases under type I interferonopathies also has important treatment implications. Historically these diseases have required multiple immunosuppressive treatments and sometimes even BMT. However, the development of JAK inhibitors has led to significant advances in treatment. New recommendations from EULAR/ACR now include using JAK inhibitors to treat PRAAS/CANDLE, SAVI, and AGS, and growing evidence suggests additional utility in other type I interferonopathies (78). In addition, several ongoing clinical trials with JAK inhibitors are being conducted in patients with SLE.

Despite the significant advances in the last decade, it remains unclear why some interferonopathies lead to systemic autoinflammatory and autoimmune disease while others present with prominent neurologic or vascular features. Additionally, JAK inhibitors have demonstrated promise in treating type I interferonopathies. However, they may be ineffective in some cases. Therefore, further work is necessary to identify novel mutations and subsequently elucidate pathophysiology to develop new therapeutic options that will improve quality of life and decrease complications of these rare diseases.
